# Simultaneous Measurement of Amyloid Fibril Formation by Dynamic Light Scattering and Fluorescence Reveals Complex Aggregation Kinetics

**DOI:** 10.1371/journal.pone.0054541

**Published:** 2013-01-17

**Authors:** Aaron M. Streets, Yannick Sourigues, Ron R. Kopito, Ronald Melki, Stephen R. Quake

**Affiliations:** 1 Department of Applied Physics, Stanford University, Stanford, California, United States of America; 2 Laboratoire d'Enzymologie et Biochimie Structurales, Centre National de la Recherche Scientifique, Gif-sur-Yvette, France; 3 Department of Biology, Stanford University, Stanford, California, United States of America; 4 Department of Bioengineering, Stanford University, Stanford, California, United States of America; 5 Howard Hughes Medical Institute, Stanford University, Stanford, California, United States of America; Tufts University, United States of America

## Abstract

An apparatus that combines dynamic light scattering and Thioflavin T fluorescence detection is used to simultaneously probe fibril formation in polyglutamine peptides, the aggregating subunit associated with Huntington's disease, *in vitro*. Huntington's disease is a neurodegenerative disorder in a class of human pathologies that includes Alzheimer's and Parkinson's disease. These pathologies are all related by the propensity of their associated protein or polypeptide to form insoluble, β-sheet rich, amyloid fibrils. Despite the wide range of amino acid sequence in the aggregation prone polypeptides associated with these diseases, the resulting amyloids display strikingly similar physical structure, an observation which suggests a physical basis for amyloid fibril formation. Thioflavin T fluorescence reports β-sheet fibril content while dynamic light scattering measures particle size distributions. The combined techniques allow elucidation of complex aggregation kinetics and are used to reveal multiple stages of amyloid fibril formation.

## Introduction

Huntington's disease is an autosomal dominant neurodegenerative disorder in a family of CAG trinucleotide repeat disorders which includes Spinobulbar muscular atrophy and six types of Spinocerebellar ataxia [1]. These genetic disorders are characterized by a CAG repeat expansion in the coding region of a mutated gene and bring about an expanded polyglutamine tract in the encoded protein. While 17–20 glutamines are typically found in the non-mutated protein, expression of huntingtin (Htt) protein with an elongated chain of greater than 35 uninterrupted glutamines (Q35) is associated with Huntington's disease (HD) [2].

Mutated huntingtin protein aggregates into insoluble, amyloid fibrils that have structural and cytotoxic similarities to aggregates associated with other neurodegenerative disorders such as Alzheimer's and Parkinson's disease [3]. All of these disorders are classified in a more general category of human pathologies termed protein deposition diseases which are characterized by the organization of soluble protein into β-sheet rich fibrils that are deposited in extracellular plaques or intracellular inclusions bodies. In the neurodegenerative subset of deposition disorders, amyloid inclusions bodies are implicated in neuronal death [4] and thus much work has been done to elucidate the nature of amyloid aggregation [3], [5]–[9].

Aggregation of polyglutamine (polyQ) peptides *in vitro* shows that glutamine repeat length dependence strongly correlates with HD pathology and age of onset [2], [9], [10]. Thais, rapid aggregation is typically observed for Q35 peptides and larger while little or no aggregation is observed with repeat lengths smaller than Q25. Additionally, a strong inverse correlation has been measured between disease age of onset and repeat length (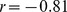) where a large part of the correlation derives from long repeat lengths (>Q60) which cause early disease onset [2]. Amyloid fibrils also display strong concentration dependent aggregation kinetics [9] which is consistent with nucleated aggregation pathways, in which fibril nucleation is the rate limiting process in amyloid formation. It has since been suggested that fibril formation is a general property of polypeptide chains [11], a conclusion which is supported by both the physical and toxic similarities between aggregates grown *in vivo* and *in vitro* [11], [12] across a wide range of peptide sequences. To the extent that amyloid fibril formation is a consequence of the physical properties of a polypeptide chain, *in vitro* characterization of the kinetics and thermodynamics of such systems can provide valuable insight into the mechanism of aggregation which would aid therapeutic inhibition strategies.

While the potential to aggregate is a characteristic of proteins associated with neurodegenerative disorders, it is not clear that mature amyloid fibrils are the toxic species in these deposition diseases [13], [14]. In HD for example small, soluble oligomers and protofibrils have been observed coexistent with fibrils both *in vivo* [15] and *in vitro* [6] and it has been postulated that these species could play a role in the cytotoxicity of huntingtin assemblies [15]. Additionally amyloid fibril formation is generally understood to proceed via a nucleated growth mechanism. Nucleated growth is an activated process in which maturation of the new phase, in this case fibril elongation, can only proceed after a stable nuclei of the new phase is formed. Nucleation requires the system to overcome an energy barrier associated with nuclei formation and thus nucleated growth is often characterized by a lag phase proceeded by rapid growth. This process has been studied in a range of amyloid systems [7], [16]–[19]. While measurement of the sigmoidal growth kinetics of fibril formation is a traditional way of extracting properties of amyloid aggregation, it has become clear that non-fibrilar species like misfolded protein monomers [3], [7] or oligomeric precursors [15], [20] play a vital role in this process. Thus a mechanistic understanding of kinetic pathways towards aggregation and identification of intermediate states and stable or metastable non-fibrilar species which coexist with peptide monomers and amyloids is a critical goal of *in vitro* aggregation studies.

Many strategies have been employed to monitor polyglutamine peptide aggregation with the goal of elucidating fibril phase behavior, including analytical ultracentrifugation [5], sedimentation assays [21], and a host of spectroscopic techniques [16], [22]–[24]. Of these spectroscopic approaches, the fluorescent dye Thioflavin T (ThT) is a popular reporter of amyloid aggregation because it demonstrates a strong shift of its fluorescent spectra upon binding to β-sheet rich fibrils [23]. The fluorescent enhancement that occurs upon ThT binding to features in the β-sheet rich structure of amyloids makes ThT fluorescence useful for *in vitro* studies of amyloid aggregation kinetics.

While ThT fluorescence is well suited to monitor β-sheet fibril formation, ThT binding assays cannot identify non-fibrilar species, interpret fibril hyperstructure, or distinguish many small fibrils from fewer large fibrils because the fluorescence associated with ThT binding only quantifies total β-sheet content. Equally, non-aggregated monomer quantification techniques like sedimentation assays cannot distinguish various species of aggregated protein. Therefore it is often informative to use multiple assays in concert, overlaying results from different techniques to reveal critical features of the aggregation pathway [7].

Dynamic light scattering (DLS) is a laser scattering technique capable of unbiased analysis of size distributions of diffusing particles in the nanometer to micron size range [25]. When coherent light rays are scattered from Brownian particles, interference causes temporal fluctuations in the detected intensity. The timescale of these temporal fluctuations correlate with diffusion time constants from which particle size is inferred. The ability to resolve multimodal size distributions and make absolute size measurements makes DLS a powerful technique for systems with heterogeneous species. DLS has been used to quantitatively study fibril formation in a range of systems from lysozyme aggregation [26], to Aβ [16] and polyglutamine aggregation [27].

**Figure 1:**
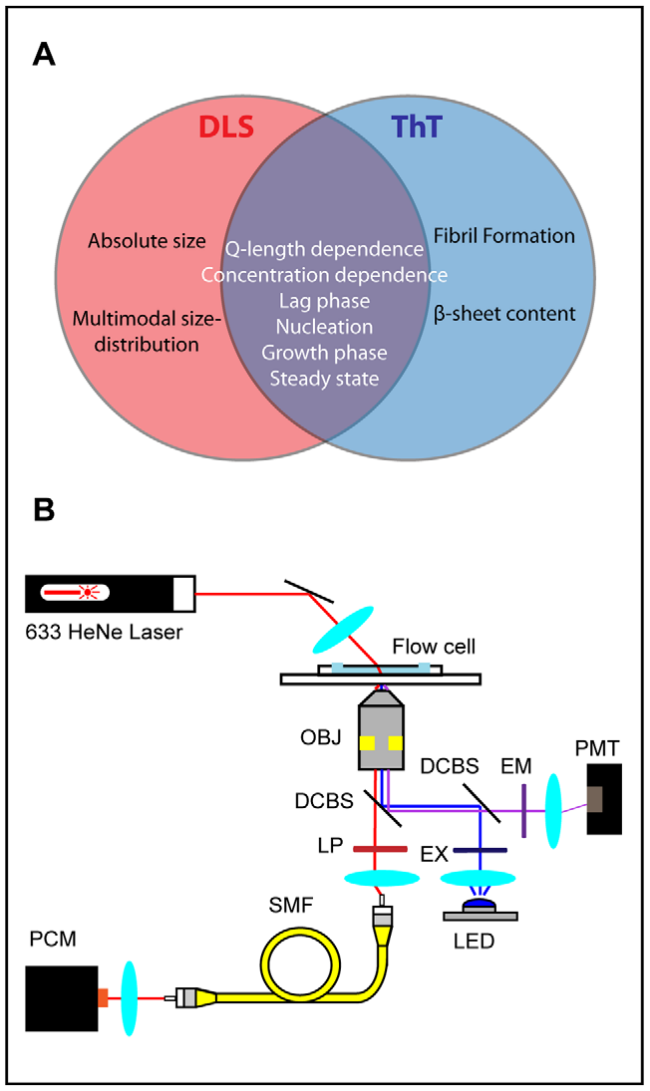
Dynamic light scattering and Thioflavin T fluorescence. (A) Dynamic light scattering and Thioflavin T comparison Venn diagram. The items within each circle summarize aspects of fibril formation which each technique is capable of measuring. (B) Schematic of DLS and ThT apparatus.

DLS is a useful complimentary technique to ThT fluorescence because in addition to detecting fibrils, it can detect diffusing species that ThT cannot, like non-fibrilar intermediates and large assemblies of fibrils. Meanwhile ThT can verify the fibrilar nature of the species which DLS resolves. The two methods are not orthogonal however as they both report some of the fundamental characteristics of polyQ aggregation, for example Q-length and concentration dependence as is shown in this work. Figure 1A summarizes the relative capabilities of the two techniques.

**Figure 2:**
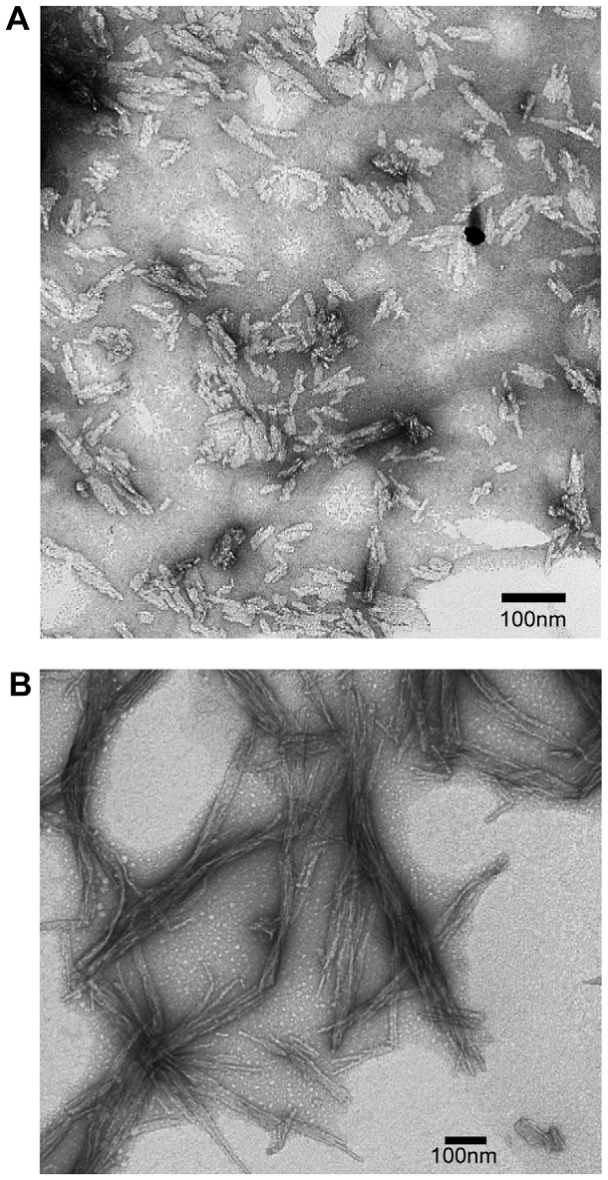
PolyQ amyloid fibrils. TEM micrograph of the products formed after TEV cleavage of the MBP from the polyQ-fusion protein complex. (A) Q45 proto-fibrilar precursors. (B) Mature Q45 fibrils.

In this work, an apparatus was built which can simultaneously perform dynamic light scattering measurements and measure ThT fluorescence in order to distinguish ThT-fibril binding events from other modes of aggregation in a single reaction (fig. 1B). The difference in the shape and respective timescales of the growth curves measured by the two assays sheds light on the various species involved in the aggregation of polyglutamine peptides. Specifically, the aggregation of polyQ peptides (Qn) was monitored upon their release from a fusion protein consisting of maltose binding protein (MBP), a tobacco etch virus (TEV) cleavage site, and a polyQ peptide chain ([MBP]-[TEVcleavage site]-[Qn]), by treatment with TEV protease. The MBP moiety within the fusion protein prevents polyQ fibrilization until TEV cleavage [28]. After cleavage, amyloid aggregation ensued and the proto-fibrilar precursors and resulting fibrils were structurally similar to those of the Htt exon 1 (fig. 2). This fusion protein system allowed us to have a consistent and controlled time-zero for aggregation reactions, in order to accurately compare independent aggregation experiments.

## Materials and Methods

### 2.1 Polyglutamine fusion complex preparation

The fusion polypeptide [His-tag]-[MBP]-[TEVcleavage site]-[Qn], where *n = 15, 25, 35, or 45*, was expressed in BL21DE3 (Stratagene, Santa Clara CA, USA). Following induction in the presence of 0.5 mM IPTG at 37°C for 2 h, the cells were sedimented and resuspended in 3 mM imidazole, 250 mM KCl, and 1 mM DTT. They were then lysed by sonication and the cell extract was spun at 30,000×g and 4°C for 30 minutes. The supernatant was filtered through 0.4 µm nitrocellulose membranes and loaded on a 10 ml Talon column and equilibrated in 3 mM Imidazole, 200 mM KCl, and 1 mM DTT. The column was washed with five bed volumes of equilibration buffer and the fusion protein was eluted using an imidazole gradient 3–200 mM. Purified [His-tag]-[MBP]-[TEVcleavage site]-[Qn] was flash frozen in liquid nitrogen and stored at −80°C. The concentration of [His-tag]-[MBP]-[TEVcleavage site]-[Qn] was determined spectrophotometrically using an extinction coefficient of 67840 M-1. cm-1 at 280 nm.

### 2.2 Dynamic Light Scattering

A dynamic light scattering apparatus was built in order to easily integrate DLS with the fluorescence excitation and detection system (fig. 1B). The DLS system is a modified version of a previously reported apparatus [29]. A 5 mW, 633 helium-neon gas laser (Melles Griot, Carlsbad CA, USA), was focused down to a 50 µm waist by a 25 mm focal length plano-convex achromatic lens (Edmund Optics, Barrington NJ, USA). The laser focal spot was positioned in the middle of a flow cell made from a disposable flow cell chamber (Grace Bio-labs, Bend OR, USA) adhered to a 1 mm thick glass microscope slide. The incident beam was positioned 30 degrees to the plane of the slide. A 10X, 10 mm focal length objective (OBJ) positioned directly beneath the flow cell collected light scattered from the sample defining a scattering vector 60 degrees to incidence. Scattered laser light was directed through a 550 nm cutoff dichroic mirror (DCBS1) (Chroma Tech, Bellows Falls VT, USA), then through a long-pass (LP) 600 nm cutoff filter (Chroma) in order to protect the detector from the fluorescence excitation field and to select light scattered from the incident beam. The coherence angle, a critical parameter for determining the signal to noise ratio in dynamic light scattering [25], was defined by a 50 µm spatial filter (Edmund Optics) positioned in a plane conjugate to the back of the objective. The intersection between the field of view of the collection optics and the laser focal region inside of the flow cell created a scattering volume of less than 300 pL. The filtered scattered light was coupled into a single mode fiber (SMF) via an NA matched fiber coupler lens (Edmund Optics). In addition to allowing a more convenient placement of the photo-detector, the single-mode fiber acted as a mode filter and improved the signal to noise of the dynamic light scattering measurement [30].

The SMF delivered scattered photons to an avalanche photo diode based photon counting module (PCM) (Perkin Elmer, Waltham MA, USA). The PCM digitized photon arrival events and output a digital pulse train representing photon counts which were counted by a National Instruments PCI counter-card and a PC. Labview software (National Instruments, Austin TX, USA) processed the raw data and computed the autocorrelation for dynamic light scattering analysis. The incident laser beam was gated by an electronic shutter so that the sample was only illuminated during data collection. In a typical experiment scattered photons were collected over sample time of 100 seconds in 50 µs bins.

Autocorrelation functions were computed using a multi-tau scheme enabling a lag-time dynamic range spanning from tens of microseconds to minutes. This dynamic range is important for aggregation studies because of the large variation in diffusional timescales between polyQ monomers and large complexes of amyloid fibrils.

For a monodisperse suspension of Brownian particles the electric field autocorrelation function 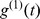 decays exponentially at a rate proportional to the translational diffusion coefficient *D*:
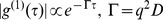
(1)where *q* is the scattering vector, 
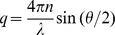
, *n* is the index of refraction of the solvent and λ is the incident wavelength. For spherical particles, the hydrodynamic radius, *R_h_*, is related to the translational diffusion coefficient by the Stokes-Einstein relation.



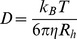
(2)
*k*_B_ is the Boltzmann constant, *T* is the absolute temperature in Kelvin, and *η* is the solvent viscosity. In suspensions of biological samples, there is typically a polydisperse distribution of diffusional timescales associated with heterogeneity in hydrodynamic radius and particle shape. This is especially true for fibrilar systems. In these cases the autocorrelation function is better represented as continuous sum of exponential decays.
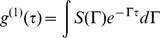
(3)


Properties of the decay distribution *S(Γ)*, like mean and variance, were computed using the method of cumulants [31], where the measured autocorrelation is fit to an expansion around the zeroth cumulant of the distribution. The mean hydrodynamic radius, <*R_h_*>, of aggregating species was calculated from *<Γ>* using equation (2) and measured values of *q*.

Cumulant analysis is typically appropriate for, monomodal distributions. When populations of diffusing particles are multimodal and have distinguishable peaks, it is informative to solve the distributions directly. CONTIN [32] was used to perform the inverse Laplace transform on equation (3) and compute the distribution *S(Γ)* using constrained regularization.

### 2.3 Thioflavin T Fluorescence

Thioflavin T (Sigma-Aldrich, St. Louis MO, USA) was dissolved in water to yield a 1 mM stock solution. This stock solution was added to polyQ aliquots before addition of TEV at a molar ratio of 10∶1 ThT:polyQ. Epi-mode fluorescence excitation and emission paths are depicted in figure 1B. ThT was excited using a high power LED (Phillips Lumiled, San Jose, CA) with a dominant wavelength of 450 nm. The excitation beam was collimated and spectrally filtered with a high-quality (HQ) excitation filter (EX) (Chroma) and then directed to the sample as depicted (fig. 1B). Fluorescent emission was collected and decoupled from laser scattering with the aforementioned dichroic beamsplitter (DCBS1). The emitted fluorescent signal was then separated from the excitation beam with a second dichroic beamsplitter (DCBS2) (Chroma) and filtered with a HQ emission filter (EM) (Chroma), before refocusing and detection with a photo-multiplier tube (PMT). The voltage output from the photo-multiplier was sampled by a Labview interfaced data acquisition card (National Instruments) at one milisecond intervals and averaged over a one second integration period. The LED could be engaged and disengaged with a digital trigger from the Labview software, similarly the HeNe laser incident beam for scattering was gated by a computer controlled shutter as described. In a typical experiment, the LED was engaged, fluorescent data was recorded, and then the LED was disengaged before proceeding to light scattering data collection. In this way any cross talk between light scattering and fluorescence channels was avoided.

### 2.4 Aggregation experiments

To monitor fibril assembly with dynamic light scattering and Thioflavin T, 30 µl of a solution containing the [MBP]-[TEV cleavage site]-[Qn] fusion protein, ThT, and TEV protease was injected into the flow cell depicted in figure 1B. The TEV protease (Invitrogen, Grand Island NY, USA), released the polyQ from the fusion protein. PolyQ assembly initiated as soon as free polyQ was generated. This solution contained a 10∶1 ratio of ThT:polyQ and 5∶1, polyQ:TEV, and was mixed immediately before injection into the flow cell. It would typically take 3 minutes to align the incident laser for optimal signal to noise in the light scattering measurements, and so the t = 0 point in recorded aggregation data sets was 4±0.5 minutes after the TEV protease is mixed with the fusion protein. Photon intensity autocorrelations were measured by DLS and ThT fluorescence intensity was recorded by the PMT every 5 minutes for 12 hours or longer. Characteristics of the aggregate size distribution were extracted from the autocorrelation post-data collection as described above. All aggregation experiments were performed at 21°C.

### 2.5 Transmission Electron Microscopy

The nature of the oligomeric species was assessed using a Jeol 1400 transmission electron microscope (Jeol Ltd., Peabody MA, USA) following adsorption of the samples onto carbon-coated 200-mesh grids and negative staining with 1% uranyl acetate. The images were recorded with a Gatan Orius CCD camera (Gatan, Pleasanton CA, USA).

## Results and Discussion

**Figure 3:**
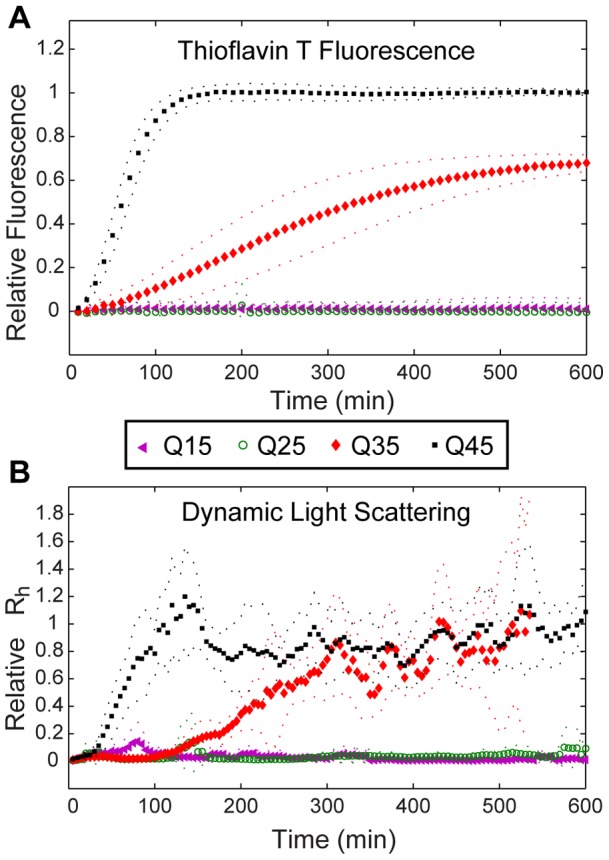
Repeat length dependent polyQ aggregation. Aggregation of Q15, Q25, Q35, and Q45 measured by ThT fluorescence (A) ([ThT] = 700 μM) and DLS (B). The data are average values of mean radius and ThT fluorescence compiled from three independent experiments. The dotted lines represent standard deviation. Both ThT fluorescence and mean hydrodynamic radius data for all glutamine repeat lengths were normalized to the steady state value of the Q45 growth curve, respectively. DLS growth curves were smoothed with a moving average filter.

Polyglutamine peptide aggregation was monitored while varying both glutamine repeat length and concentration. Polyglutamine peptides including tracts of about 35 or more continuous glutamines demonstrate relatively rapid assembly into amyloid fibrils and likewise, tracts of greater than 35 glutamines in the huntingtin exon 1 lead to HD pathogenesis. Aggregation was initiated by TEV-mediated release of the polyQ peptide from the MBP-TEV-polyQ fusion complex for Q45, Q35, Q25, and Q15 (70 µM). Thioflavin T fluorescence and dynamic light scattering both confirmed this critical Q-length reporting almost complete fibril assembly in a 12 hour period for Q45 and Q35 while reporting no ThT binding or any shift in mean diffusion coefficient in the same period for Q25 and Q15 (fig. 3). In figure 3A ThT fluorescence was normalized to the mean fluorescence signal of mature Q45 fibrils. During this 12 hour period, both the TEV protease and the MBP complex were stable reporting no increase in ThT fluorescence, mean hydrodynamic radius, or time-averaged scattered intensity (figs. S1 and S2).

The ThT growth curves for Q45 and Q35 are sigmoidal showing a dependence on time, which suggests that the assembly process is cooperative. Q35 demonstrates elongated lag and growth phases compared to Q45. Additionally, the fluorescent yield of ThT bound to mature Q35 fibrils is almost 70% of that recorded for Q45 indicating fewer β-sheet subunits in the total population. Growth kinetics reported by dynamic light scattering reveal the same Q-length dependence as expected but the growth curves take a slightly different shape than those measured with ThT (fig. 3B). Q35 in particular displayed a relatively long inactive lag phase before the growth of <*R_h_*> just after 100 minutes. Here mean hydrodynamic radius is calculated by use of the method of cumulants and normalized to the steady state Q45 values. Contributions to the amplitude of the static scattering signal from TEV protease and MBP-Q45 monomers are about 8×10^3^ photons/sec and 10^4^ photons/sec respectively. These magnitudes are 50 to 70 times smaller than the contributions from mature fibrils. As the polyQ peptides begin to aggregate the static, time-averaged scattering signal increases above the baseline scattering from these non-aggregating molecules (fig. S2). The background dynamic light scattering signal from TEV and MBP complex contributes to a baseline size distribution which centers around 10 nm (fig. S3). As aggregating polyQ peptide assemblies become larger than this 10 nm background, they become distinguishable with DLS (fig. S1B).

In these dynamic light scattering measurements all particles in the scattering volume are approximated by spheres. Mature fibrils, in fact, often display a rod-like or fibrous morphology when imaged with electron microscopy (fig. 2) or atomic force microscopy and studies have used such techniques to measure fibril length or interpret DLS data [16], [26]. Here, since we are observing many species simultaneously, the spherical approximation is used to avoid bias in autocorrelation fitting caused by *a priori* assumptions about particle shape. It should be noted however that in addition to this approximation the ability to accurately extract particle size with dynamic light scattering diminishes with increased aggregate size, conformational heterogeneity, and scattering fluctuations caused by increased rotational diffusion. Additionally, as large particles sediment within the scattering volume and cease to diffuse they become static scattering centers and contribute to noise in the DLS measurement.

Nucleated aggregation is a stochastic process and thus growth rates and lag times can vary up to 10 or 20 percent from experiment to experiment (fig. 3). This intrinsic variation makes it difficult to compare results between different aggregation assays when they are performed on separate aggregation experiments. Using the combined system, the same aggregating sample can be examined with both DLS and ThT simultaneously. This approach ensures that discrepancies between the two measurement techniques are real and not caused by intrinsic variation in aggregation kinetics from one experiment to the next.

**Figure 4:**
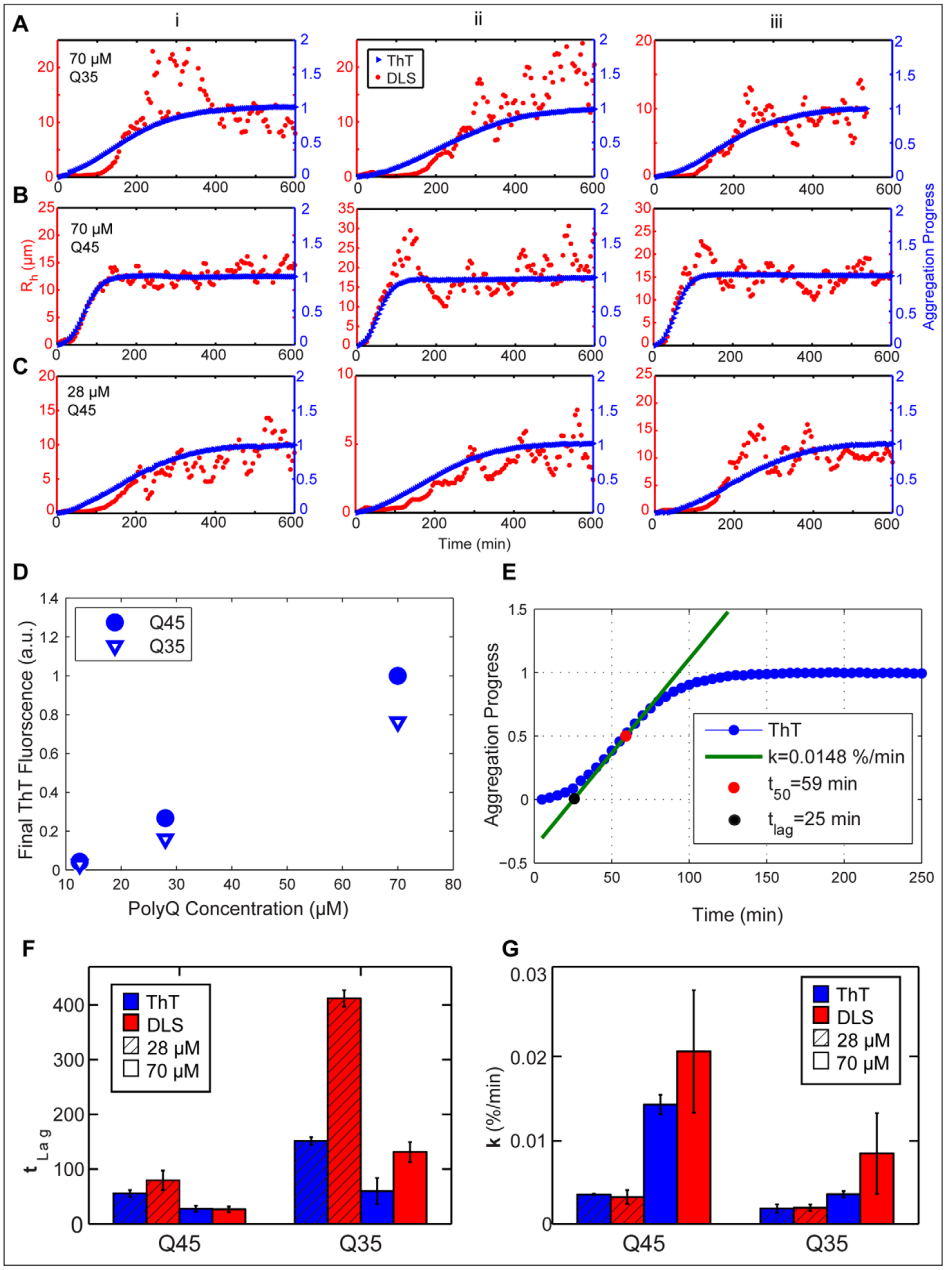
Simultaneous measurement of aggregation by DLS and ThT fluorescence. Repeat length and concentration dependent aggregation measured simultaneously by DLS and ThT fluorescence for (A) 28 µM Q45 (B) 70 µM Q45 and (C)70 µM Q35. Columns i, ii, and iii display repeat experiments. The DLS data are smoothed with a moving average filter. (D) Final ThT fluorescence normalized to 70uM Q45. (E) Calculation of T_lag_ and K_1/2_ from sigmoidal fit. (F) T_lag_ and (G) K_1/2_ extracted from sigmoidal fits to DLS and ThT data.

ThT binding and mean hydrodynamic radius evolution were measured simultaneously for Q35 and Q45 at three concentrations, 12 µM, 28 µM and 70 µM. The 12 µM reaction produced almost negligible increase in mean hydrodynamic radius for both Q45 and Q35 (not shown). Figure 4 compares aggregation measured by both ThT and DLS for 70 µM Q35, 70 µM Q45 and 28 µM Q45. At the same concentration, Q35 aggregates slower than Q45 as expected, but the lag phase of the growth curve of the 70 µM Q35 sample as measured by DLS is longer than that measured by ThT (fig. 4A). ThT binding seems to ensue almost immediately after TEV cleavage whereas <*R_h_*> does not increase until just after 100 minutes. A similar disparity between DLS and ThT is observed in the more slowly aggregating 28 µM Q45 sample.

The particles observed by DLS during the lag phase of the slowly aggregating samples tend to have <*R_h_*> on the order of 10 nm (fig. S3). In these experiments, the increase of ThT fluorescence which coincides with the apparent lag phase of the DLS aggregation curves implies that the dye is binding to some fibrilar structure during this period. This elongating fibrilar species must have a diffusion coefficient equivalent to or greater than a 10 nanometer particle, or else the elongation would be measured by light scattering as well. This indirectly suggests that the smallest ThT binding β-sheet unit has a hydrodynamic radius less than or equal to ten nanometers, a hypothesis supported by previous studies which conclude that the smallest ThT binding unit is cross β-sheet fibrils made of five or more β-strands [33].

This disparity between <*R_h_*> and ThT binding does not occur in the rapidly aggregating 70 µM Q45 sample. Instead the two assays concomitantly report aggregation reaching a steady state plateau in both β-sheet content and <*R_h_*> simultaneously (fig. 4B). ThT reveals higher steady-state fluorescence with increasing concentration (fig. 4D). Equally, DLS reports a slightly larger steady-state <*R_h_*> in the 70 µM Q45 reaction than the 28 µM while the 12 µM sample displays almost no increase in <*R_h_*> (not shown) confirming that fibril elongation is stunted at lower concentrations. ThT fluorescence alone only measures total β-sheet content, and is blind to the way in which β-sheets are distributed within fibrils. Thus without the complimentary DLS information it would be impossible to discern whether the lower relative ThT fluorescence measured in the less concentrated reactions was caused by shorter fibrils or fewer fibrils.

70 µM Q35 aggregates slower than the 70 µM Q45 as previously noted and like 28 µM Q45 reveals a discrepancy between ThT binding and <*R_h_*> evolution (fig. 4C). Features of the aggregation kinetics were quantified by fitting growth curves to the sigmoid function
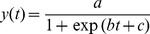
(4)and extracting the lag time, *t_lag_*, and transition slope, *k_1/2_* defined in figure 4E. The Q-length and concentration dependence of polyQ fibril aggregation is corroborated by the observation that *t_lag_* decreases and *k_1/2_* increases as concentration and Q-length are increased (figs. 4F and 4G). This is observed by both ThT fluorescence and dynamic light scattering. The difference in shape between aggregation curves measured by DLS and ThT is summarized by the observation that in the slowly aggregating polyQ samples, specifically the 28 µM Q45 and 70 µM Q35, DLS reports a longer *t_lag_* and a greater *k_1/2_* than ThT. This can be interpreted to mean that ThT measures a faster nucleation rate, but a slower elongation rate than DLS.

**Figure 5:**
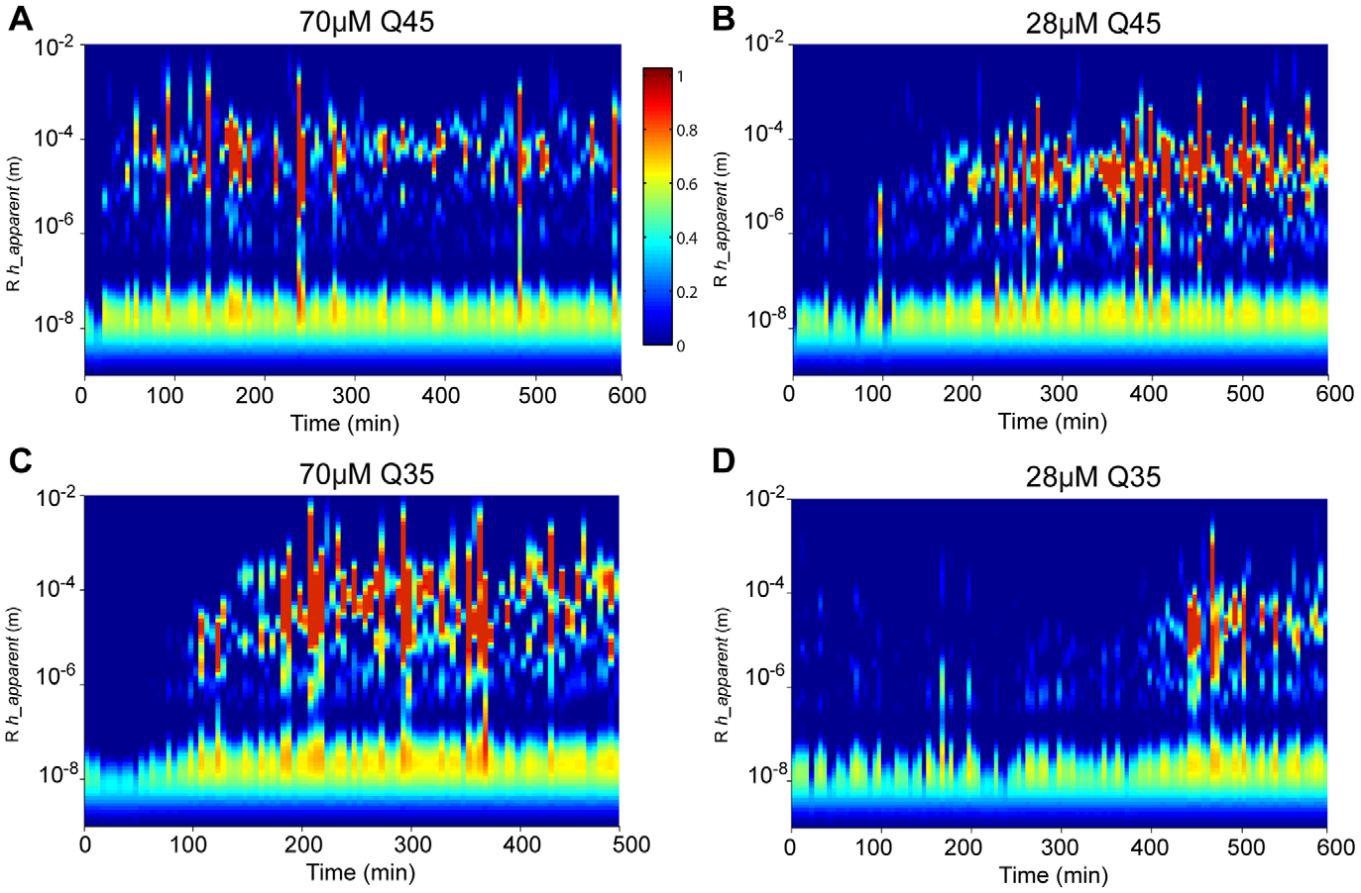
Size distribution evolution during aggregation. CONTIN size distributions of aggregating polyQ. (A) 70 uM Q45 (B) 28 uM Q45 (C) 70 uM Q35 (D) 28 uM Q35.

This difference in shape must be caused by the fundamental difference between the species which ThT and DLS respectively observe. The magnitude of the ThT fluorescent signal reports the total number of minimal dye-binding β-sheet subunits. DLS measures the size distribution of diffusing particles and the signal is dominated by populations that exhibit the most scattering, specifically large or abundant species. In order to gain a more complete understanding of the species which DLS detects, CONTIN was used to solve equation (3) for *S(Γ)* and extract particle size distributions from the light scattering data from aggregating Q45 and Q35 at 70 µM and 28 µM (fig. 5). CONTIN analysis revealed the evolution of multimodal distributions of scattering particles over the course of the aggregation reaction.

As the shape of aggregate particles diverges from the spherical approximation, hydrodynamic radius becomes a less accurate description of the size of the aggregates. Instead it is useful to understand aggregate size in terms of *R_h_*__apparent_ which is defined here as the hydrodynamic radius of a sphere with equal translational diffusion coefficient. Observation of the distribution of *R_h_*__apparent_ in time reveals the emergence of a population of large diffusing particles (fig. 5) which coincides with the growth phase of <*R_h_*> reported by cumulant analysis in figure 4. The magnitude of the light scattering signal from mature aggregates is in fact dominated by photons scattered from these larger species at about 5×10^5^ photons/sec. This is apparent in the larger relative amplitudes of the peaks at the higher region of the particle size distributions in figure 5. The method of cumulants attempts to fit a single peak to the whole particle size distribution, including all species of scattering particles. The smaller species, while scattering fewer photons, still give a strong contribution to the decay rate of the autocorrelation function. Thus while the temporal features of the particle size distribution evolution measured by CONTIN track with the mean radius measured by the method of cumulants the particle size measurement in figure 4 is only an average value. However CONTIN is also susceptible to noisy measurement caused by non-spherical fibrils which contain more complex diffusion characteristics including rotational diffusion and inter-fibrilar motion. Additionally at late time points during assembly, the static background scattering signal from sediment particles causes an ill-defined baseline in the measured autocorrelation functions. This is can be interpreted by CONTIN as contributions from slowly decaying time components of the autocorrelation function biasing the measurement towards larger radii. Nonetheless the CONTIN distributions offer insight into the polydispersity of various species measured by DLS. In accordance with the cumulant measurements, the emergence of the large species revealed by CONTIN analysis do not coincide with the rapid fibril growth measured by ThT fluorescence.

**Figure 6:**
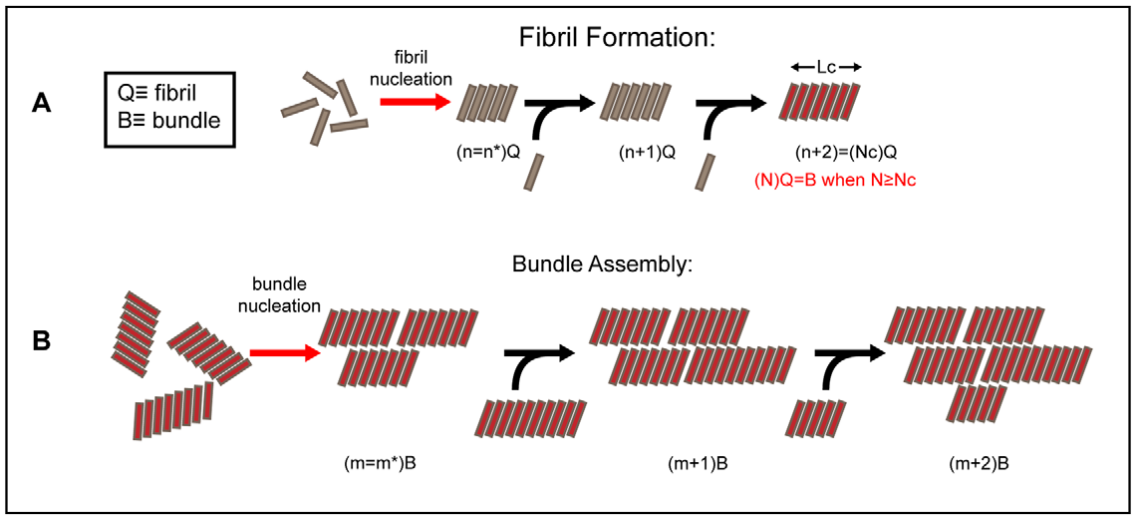
Model for fibril and bundle formation. (A) Fibril nucleation and growth. n* is the number of polyQ monomers needed to form the critical nucleus for fibril nucleation. Elongation proceeds by monomer addition. When the length of the fibril reaches a critical length *L_c_*, which occurs when the number of monomers which make up the fibril reaches *N_c_*, the fibril can begin to assemble into fibril bundles. (B) Bundle nucleation and growth. Fibrils of length *L≥L_c_* are the minimal unit for bundle assembly. A critical bundle nucleus is formed when m* bundles associate. Bundle growth ensues as individual bundles of *L≥L_c_* attach to the bundle nucleus.

To understand the nature of the discrepancy between ThT and DLS, we consider a simplified model of fibril growth [8] in which there are only two species; polyQ monomers and a distribution of linear fibrils centered on a mean length of *n* monomers. In this model nucleation ensues and fibrils begin to elongate by monomer addition after a lag phase associated with the fibril nucleation rate (fig. 6A). In such a case where the only aggregate growth mechanism – and thus the only cause for decrease in translational diffusion coefficient or an increase in *R_h_*__apparent_ – comes from fibril elongation via the formation of new β-sheet elements, CONTIN analysis would reveal a heterogeneous particle distribution peaked at an *R_h_*__apparent_ corresponding to the mean fibril length, and the mean of this distribution would track with the ThT fluorescence curve. This is observed during the rapid aggregation of 70 µM Q45 (fig. 4B). For cases of slower aggregation however, ThT fluorescence kinetics diverged from the kinetics of particle size distribution measured by DLS and thus implies another mechanism by which <*R_h_*> increases. If there were an abundant non-fibrilar intermediate state along the kinetic trajectory to fibril formation, DLS would report the presence of these species as an increased <*R_h_*> or an independent peak in the particle size distribution, *preceding* ThT mediated fluorescent enhancement. Instead, in the more slowly aggregating samples, 28 µM Q45 and Q35 and 70 µM Q35, ThT reported fibrils that emerge first, and are then succeeded by a rapid increase in <*R_h_*>. The early ThT binding fibrils must be smaller than the 10 nm baseline in <*R_h_*> measurement and so in this case DLS only detects species with a larger *R_h_*__apparent._ These data suggest that the large species reported by DLS require the presence of fibrils to form. It is therefore possible that these species are in fact a higher-order cluster of amyloid fibrils. It should be noted that the data does not rule out the possibility of non-fibrilar intermediate species which precede fibril formation. These proto-fibril intermediates however must then be smaller than the 10 nm baseline in the <*R_h_*> measurements.

Amyloid fibrils are often observed in tightly bunched plaques as seen in figure 2. It is possible that when individual fibrils reach a certain length, it becomes energetically favorable for fibrils to assemble into higher order structures. The formation kinetics of these large fibril bundles would thus be directly dependent on fibril nucleation and elongation rates.

A qualitative model is used to illustrate fibril bundling that is based on a classical single-step nucleation scheme (fig. 6). Two critical parameters associated with the fibril bundle nucleation energy barrier are defined. The critical fibril length *L_c_*, is defined as the mean fibril length that is required for fibril bunching. In other words it is energetically favorable for fibrils shorter than *L_c_* to exist in an isolated state while fibrils longer than *L_c_* have the potential to assemble into bundles. Fibrils of length *L* contain *N* polyQ monomers so *N_c_* is the critical number of monomers in a fibril necessary for fibril bundling. A critical fibril number for bundling m* is also defined. m* defines the size of the critical nucleus for bundle formation and is analogous to the critical nucleus size for fibril nucleation n*. Fibril bundles made up of *m*<m* fibrils have a higher probability to disassociate into individual fibrils, whereas fibril bundles consisting of *m* >m* fibrils surmount the nucleation energy barrier and will recruit more fibrils for bundle growth. Thus the nucleation of fibril bundles is governed by two parameters, the rate that fibrils reach length *L*> *L_c_*, and the rate that fibrils of *L≥ L_c_* bundle together. This bundling rate is inherently dependent on the total number of fibrils with *L*> *L_c_*.

In this qualitative model, nucleation and growth of fibril bundles is directly dependent on fibril elongation. In aggregating solutions of 28 µM Q45 and 70 µM Q35, fibril elongation proceeds slowly, so there is a lag time of about 100 minutes or more required before a sufficient population of fibrils reach *L_c_*. Only then do we see fibril bundling reported by DLS. In this model it is necessary that bundling does not interfere with fibril elongation, thus the ThT binding curves coincide directly with fibril elongation which continues uninterrupted throughout the aggregation reaction.

In the case of 70 µM Q45, fibril elongation is so rapid, as confirmed by ThT fluorescence, that a large population of fibrils quickly reach the critical length *L_c_* while other fibrils are still being formed and elongated. Additionally individual fibrils within the large fibril assemblies may still be undergoing elongation. This explains the simultaneous increase of ThT fluorescence and <*R_h_*> in figure 4B. Particle size distributions also reveal very heterogeneous populations after the initial increase in *R_h_*__apparent_, suggesting a spectrum of fibril structures ranging from single fibrils to the largest fibril assemblies (fig. 5). The final population is a heterogeneous distribution of fibrils and fibril bundles, as demonstrated on the electron micrograph (fig. 2).

## Conclusion

*In vitro* studies of protein and polypeptide aggregation have shown that amyloid fibril formation is an intrinsic property of a large set of amino acid sequences. While the thermodynamic environment *in vivo* is drastically different from that used throughout this study, *in vitro* approaches nonetheless provide insight into the various thermodynamic states accessible by aggregating polypeptides as well as elucidate nucleation kinetics and mechanisms. Most *in vitro* assays however are inevitably limited in their ability to probe comprehensive aspects of fibril formation. The apparatus presented here combines two techniques which measure protein aggregation, dynamic light scattering and Thioflavin T fluorescence, for simultaneous analysis of the same aggregating sample. Applying the techniques simultaneously allowed comparison of size distributions generated by DLS and β-sheet content measured by ThT while avoiding intrinsic variation in aggregation kinetics from experiment to experiment caused by stochastic nucleation.

The observation that fibril size distribution does not consistently follow the same kinetic evolution as β-sheet content suggests the existence of multiple aggregate states of the polyQ peptides including higher order states of fibrilar assemblies. Fibril bundling may simply be a thermodynamic byproduct of the nature of the interaction of polyQ molecules. It may also be that fibril bundling is a second order mechanism intended to localize all potentially toxic species and strengthen mature fibrils in order to minimize breakage.

In understanding all of these possible aggregation pathways it is important to be able to distinguish β-sheet fibrils from non-fibrilar aggregates. Much work has been focused on the role of oligomeric precursors to fibril formation [6], [7], [15] and the ability to probe oligomers and fibril formation *in vitro* is of particular interest. The apparatus described here can be applied to such studies, enabling simultaneous measurement of non-fibrilar precursors and elongating fibrils. More generally, a tool is presented for the investigation of amyloid forming peptides, like polyQ and Aβ, the amyloid inducing peptide in Alzheimer's disease, or any protein deposition disorder involving peptides which form fibrils that bind Thioflavin T.

## Supporting Information

Figure S1 **TEV and MBP stability.** ThT fluorescence (A) and DLS measurements (B) of the beta-sheet content and mean hydrodynamic radius of a single Q45 (black filled circles) and Q35 (black open circles) assembly reaction at 70 μM compared with solutions containing only MBP-Q45 complex (blue stars) and TEV protease (red triangles). All measurements are normalized to their respective initial values.
(EPS)

Figure S2 **Time-averaged static scattering of Q45 assembly.** Absolute time-averaged scattering signal during the same experiments described in figure S1.
(EPS)

Figure S3 **TEV and MBP-complex particle size distributions.** Intensity weighted particle size distributions measured by CONTIN of MBP-polyQ complex (blue stars) and TEV protease (red triangles) which add to the background in DLS measurements.
(EPS)
